# Multiple or Single Endocrine Abnormalities Associated With Immune Checkpoint Inhibitors

**DOI:** 10.1210/clinem/dgaf347

**Published:** 2025-06-12

**Authors:** Fumika Kamitani, Yuichi Nishioka, Hiroki Nakajima, Yukako Kurematsu, Sadanori Okada, Tomoya Myojin, Tatsuya Noda, Tomoaki Imamura, Yutaka Takahashi

**Affiliations:** Department of Diabetes and Endocrinology, Nara Medical University, Kashihara, Nara 634-8522, Japan; Department of Public Health, Health Management and Policy, Nara Medical University, Kashihara, Nara 634-8521, Japan; Department of Diabetes and Endocrinology, Nara Medical University, Kashihara, Nara 634-8522, Japan; Department of Diabetes and Endocrinology, Nara Medical University, Kashihara, Nara 634-8522, Japan; Department of Diabetes and Endocrinology, Nara Medical University, Kashihara, Nara 634-8522, Japan; Department of Public Health, Health Management and Policy, Nara Medical University, Kashihara, Nara 634-8521, Japan; Department of Public Health, Health Management and Policy, Nara Medical University, Kashihara, Nara 634-8521, Japan; Department of Public Health, Health Management and Policy, Nara Medical University, Kashihara, Nara 634-8521, Japan; Department of Diabetes and Endocrinology, Nara Medical University, Kashihara, Nara 634-8522, Japan

**Keywords:** immune checkpoint inhibitor, multiple endocrine abnormalities, thyroiditis, hypophysitis, diabetes

## Abstract

**Background:**

Immune checkpoint inhibitors (ICIs) are associated with various endocrine abnormalities. However, their underlying pathophysiology remains unclear. We investigated the effect of multiple endocrine abnormalities on the overall survival (OS) of patients treated with ICIs.

**Methods:**

In total, 12 978 patients who received ICIs between 2014 and 2022 were investigated using the DeSC Japanese administrative claims database. Endocrine abnormalities were defined by each hormone replacement therapy, including levothyroxine, hydrocortisone, and insulin, in which it is difficult to distinguish central or primary hormone defect. Also, only patients with hypothyroidism after thyroiditis were included. Type 1 diabetes was additionally defined by the name of the disease and strict self-injection fees. Regression analyses were performed to identify risk factors for endocrine abnormalities and the effect of endocrine abnormalities on OS, adjusting for confounders including the number and duration of ICI administrations.

**Results:**

Single and multiple endocrine abnormalities were observed in 12.0% and 1.4% of patients, respectively. The most common combination was hypothyroidism and adrenal insufficiency (1.3%). Kaplan–Meier analysis indicated better survival in patients with multiple and single endocrine abnormalities than in those without (*P* < .01). Multivariable analysis revealed lower mortality in patients with multiple and single endocrine abnormalities [adjusted hazard ratio (aHR) 0.39; 95% confidence interval (CI), 0.28-0.54, *P* < .01; aHR 0.65; 95% CI, 0.5-80.72, *P* < .01, respectively) than in those without. Mortality was significantly lower with multiple abnormalities than with single (aHR 0.56; 95% CI, 0.39-0.79, *P* < .01).

**Conclusion:**

The development of multiple endocrine abnormalities in patients treated with ICIs is associated with improved survival compared with that of patients with a single abnormality.

Immune checkpoint inhibitors (ICIs) have substantially improved the clinical outcomes in many types of cancers (1). The number of patients receiving ICIs has increased in recent years. Immune-related adverse events (irAEs) are often associated with ICIs (2, 3).

Endocrine abnormalities, especially thyroiditis and hypophysitis, are frequently observed in patients treated with ICIs (4, 5). Interestingly, several cohort studies have reported that their development is associated with favorable tumor response and prolonged survival (6, 7). Endocrine abnormalities differ from other irAEs in terms of treatment; pharmacological steroid treatment is not appropriate (8) because these abnormalities are generally irreversible, but they can be treated with replacement therapy for deficient hormones (5) While there are several reports (9-11) and reviews (12, 13) regarding cases of multiple endocrine abnormalities, the precise epidemiology of these irAEs and their effect on prognosis (survival) remain unclear. Therefore, we conducted a large retrospective cohort study using a Japanese administrative claims database of 12 million individuals to determine the precise incidence, risk factors, and effect on survival of single and multiple endocrine abnormalities. In this condition, we defined endocrine abnormalities based on the replacement therapy; therefore, it is difficult to distinguish central or primary hormone defects. Also, only patients with hypothyroidism after thyroiditis were included. However, using these definitions enabled us to analyze this large-scale administrative claims database, including the effect on survival.

## Materials and Methods

### Study Population

Patients who received ICIs between April 2014 and February 2022 were identified among 12.4 million individuals in the Japanese administrative claims database (DeSC database). The constitution of the DeSC database population is reportedly similar to the entire population of Japan; therefore, it is suggested that the database can be used for representative medical epidemiology (14). The ICIs included anti-programmed cell death 1 (PD-1) monotherapy (nivolumab, pembrolizumab), anti-programmed cell death ligand 1 (PD-L1) monotherapy (atezolizumab, durvalumab, and avelumab), anti-cytotoxic T-lymphocyte antigen 4 (CTLA-4) monotherapy (ipilimumab), and combination and sequential therapies. Combination therapy with anti-PD-1 and anti-CTLA-4 refers to the simultaneous administration of these agents, whereas sequential therapy with anti-PD-1 and anti-PD-L1 refers to the administration of these agents at different times during the observation period. All patients were aged ≥18 years. To exclude preexisting ICI treatment and ICI-related endocrine abnormalities and to ensure an observation period, patients who had <180 days of observation period prior to the first ICI administration and patients who had <180 days of observation period after the first ICI administration, other than because of death, were excluded. In the analysis of each endocrine abnormality, we excluded patients who had been treated for endocrine abnormalities before ICI treatment (Supplementary Fig. S1) (15). This study was approved by the Ethics Review Committee of Nara Medical University (approval number: 1123-8); and the requirement for informed consent was waived because all data were anonymized.

### Outcome and Disease Definition

The main outcome of this study was the development of a new endocrine abnormality after ICI administration. Because disease names such as hypophysitis and thyroiditis often do not reveal the precise pathological conditions in claims databases, we defined endocrine abnormalities by prescription for each replacement therapy. Hypothyroidism was defined as the diagnosis of hypothyroidism and treatment with levothyroxine. It is difficult to accurately distinguish between primary and secondary hypothyroidism using the administered disease codes; therefore, this definition includes both hypothyroidism due to thyroiditis and secondary hypothyroidism due to hypophysitis. Adrenal insufficiency was defined as hydrocortisone treatment. Again, because it is difficult to accurately distinguish between primary and secondary adrenal insufficiency using the administered disease codes, this definition includes hypoadrenalism due to both hypophysitis and adrenalitis. Type 1 diabetes mellitus (T1DM) was defined by a disease code of T1DM and prescription of insulin with a self-injection fee for T1DM, which is strictly applied for T1DM in the Japanese insurance system (16, 17). The date of onset was defined as the first administration of levothyroxine, first administration of hydrocortisone, and instructional charges for T1DM, respectively. Death was defined as a transcribed death in the claims data. Evaluation of patient characteristics included assessment of the updated Charlson Comorbidity Index score (18) as a short-term prognostic score.

### Statistical Analysis

The χ^2^ test was used for statistical analysis of independence and comparison of frequencies. Logistic regression analysis was performed with age, sex, ICI type, tumor type, and Charlson Comorbidity Index score as covariates, to identify the risk factors for the development and characteristics of each endocrine abnormality. Median overall survival (OS) was estimated using the Kaplan–Meier method. When the median OS was not reached, the 1-year survival rate was applied for evaluation. To exclude immortal time bias, which could cause the error in estimating the association between the exposure and the outcome that results from misclassification or exclusion of time intervals (19), we used a Cox model adjusted to include the number and duration of ICI administration (covariates: age, sex, ICI type, number of ICI administrations, duration, cancer type, comorbidity score, ICI-related hypothyroidism, adrenal insufficiency, and T1DM) to evaluate the effect of endocrine abnormalities on survival. SPSS (IBM SPSS Statistics 28.0.1, USA) was used for all statistical analyses. Statistical significance was defined as *P* < .05.

## Results

### Patient Characteristics and Incidence of Each Endocrine Abnormality Associated With ICI

We identified 12 978 patients treated with ICIs, with a median follow-up period of 356 days (interquartile range, 216-596 days). The median age of patients treated with ICI was 73 years (interquartile range, 68-78), and 74.6% were male (Supplementary Tables S1 and S2) (15). The most common type of ICI was anti-PD-1 monotherapy (75.5%), followed by anti-PD-L1 monotherapy (17.9%) and combination therapy with anti-PD-1 and anti-CTLA-4 (3.4%). Non–small cell lung cancer was the most common cancer (52.7%), followed by gastric cancer (22.3%; Supplementary Table S1) (15).

**Figure 1.**
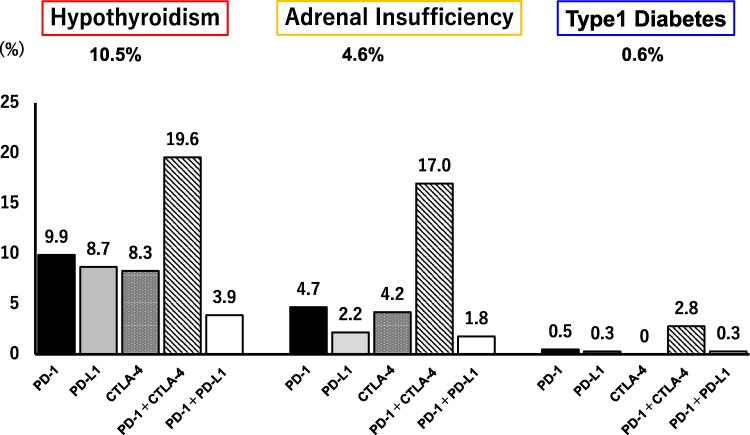
Incidence of each endocrine abnormality associated with ICI treatment. The incidence of endocrine abnormalities, including hypothyroidism, adrenal insufficiency, and type 1 diabetes, among all patients administered ICI is indicated at the top of the figure. The incidence of each ICI subgroup is also presented. Abbreviations: CTLA-4, cytotoxic T lymphocyte antigen 4; ICI, immune checkpoint inhibitor; PD-1, programmed cell death 1; PD-1 + CTLA-4, combination therapy with anti-PD-1 and anti-CTLA-4; PD-1 + PD-L1, sequential therapy with anti-PD-1 and anti-PD-L1; PD-L1, programmed cell death ligand 1.

Hypothyroidism, adrenal insufficiency, and T1DM were observed in 10.5%, 4.6%, and 0.6% of patients, respectively (Fig. 1). In terms of ICI type, all endocrine abnormalities were most prevalent in patients who received combination therapy with anti-PD-1 and anti-CTLA-4. The incidence of hypothyroidism was 19.6% in the combination therapy, 9.9% in anti-PD-1 therapy, 8.7% in anti-PD-L1 therapy, 8.3% in anti-CTLA-4 therapy, and 3.9% in sequential therapy of anti-PD-1 and PD-L1. The incidence of adrenal insufficiency was 17.0% in combination therapy, 4.7% in anti-PD-1 therapy, 2.2% in anti-PD-L1 therapy, 4.2% in anti-CTLA-4 therapy, and 1.8% in sequential therapy of anti-PD-1 and PD-L1. T1DM occurred in 2.8% of patients in combination therapy, 0.5% in anti-PD-1 therapy, 0.3% in anti-PD-L1 therapy, and 0.3% in sequential therapy of anti-PD-1 and PD-L1, and no patients developed T1DM in anti-CTLA-4 monotherapy (Fig. 1, Supplementary Table S2) (15).

### Factors Associated With the Development of Each Endocrine Abnormality

**Figure 2.**
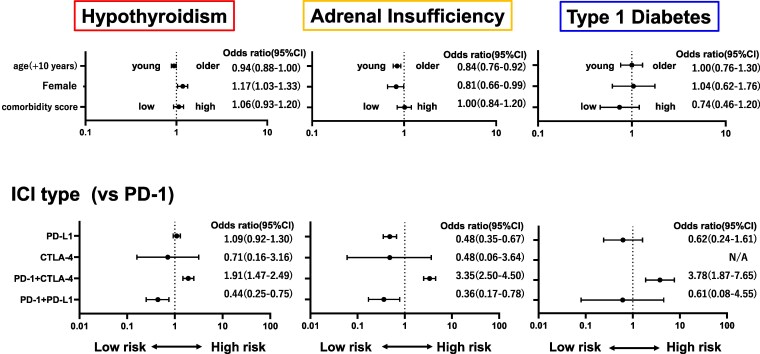
Logistic regression analysis of the risk factors for the development of endocrine abnormalities associated with ICI treatment. Multivariate analysis was performed using age (every 10 years), sex, ICI type, tumor type, and comorbidity scores as covariates. The comorbidity score was determined using the Charlson comorbidity score, and the median score for the study patients was 6; comparison was made between scores ≥6 or <6. Abbreviations: CTLA-4, cytotoxic T lymphocyte antigen 4; ICI, immune checkpoint inhibitor; PD-1, programmed cell death 1; PD-1 + CTLA-4, combination therapy with anti-PD-1 and anti-CTLA-4; PD-1 + PD-L1, sequential therapy with anti-PD-1 and anti-PD-L1; PD-L1, programmed cell death ligand 1.

The multivariate analysis demonstrated that female sex [odds ratio (OR), 1.17; 95% confidence interval (CI), 1.03-1.33; *P* = .02] and combination therapy of anti-PD-1 and anti-CTLA-4 (compared with anti-PD-1 monotherapy, OR, 1.91; 95% CI, 1.47-2.49; *P* < .01) were associated with an increased risk for developing hypothyroidism (Fig. 2). Conversely, sequential therapy of anti-PD-1 and anti-PD-L1 was associated with a decreased risk of hypothyroidism (compared with anti-PD-1 monotherapy, OR, 0.44; 95% CI, 0.25-0.75; *P* < .01). An increased risk of developing adrenal insufficiency was observed in younger patients (OR, 0.84; 95% CI, 0.76-0.92; *P* < .01), male patients (OR, 0.81; 95% CI, 0.66-0.99; *P* = .04), and those receiving combination therapy of anti-PD-1 and anti-CTLA-4 (compared with anti-PD-1 monotherapy, OR, 3.35; 95% CI, 2.50-4.50; *P* < .01). Anti-PD-L1 monotherapy (compared with anti-PD-1 monotherapy, OR, 0.48; 95% CI, 0.35-0.67; *P* < .01) and sequential therapy of anti-PD-1 and anti-PD-L (compared with anti-PD-1 monotherapy, OR, 0.36; 95% CI, 0.17-0.78; *P* < .01) were associated with a decreased risk of adrenal insufficiency. An increased risk for developing T1DM was associated with combination therapy of anti-PD-1 and anti-CTLA-4 (compared with anti-PD-1 monotherapy, OR, 3.78; 95% CI, 1.87-7.65; *P* < .01) (Fig. 2). Preexisting hypothyroidism (OR, 2.54; 95% CI, 1.27-5.07; *P* < .01) was associated with an increased risk of T1DM (Supplementary Table S3) (15).

### Incidence and Characteristics of Multiple Endocrine Abnormalities

**Figure 3.**
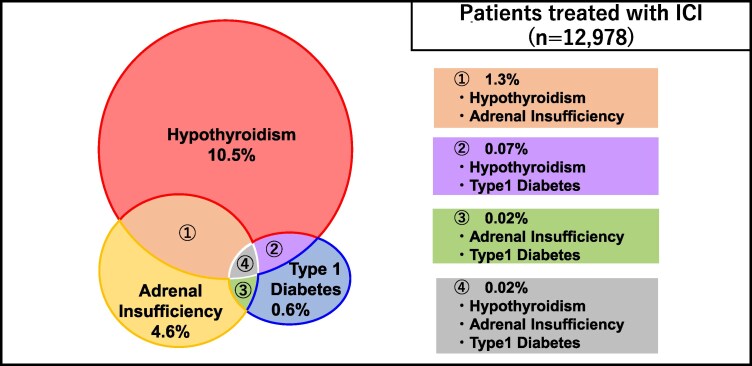
Incidence of endocrine abnormalities associated with ICI treatment. The incidence and comorbidity rates of each endocrine abnormality in patients treated with ICI are presented. Among the 12 978 patients who received ICIs, the most common combination of patients with multiple endocrine abnormalities was hypothyroidism and adrenal insufficiency (n = 170, 1.3%), with 0.07% (n = 9) having hypothyroidism and T1DM, 0.02% (n = 2) having adrenal insufficiency and T1DM, and 0.02% (n = 3) having hypothyroidism, adrenal insufficiency, and T1DM. Abbreviations: ICI, immune checkpoint inhibitor; T1DM, type 1 diabetes mellitus.

Among the 12 978 patients who received ICIs, single endocrine abnormalities occurred in 12.0% and multiple endocrine abnormalities in 1.4%. The most common combination of endocrine abnormalities was hypothyroidism and adrenal insufficiency (1.3%), followed by hypothyroidism and T1DM; adrenal insufficiency and T1DM; and hypothyroidism, adrenal insufficiency, and T1DM (0.07%, 0.02%, and 0.02%, respectively) (Fig. 3). That is, 1 of 7 patients with hypothyroidism developed adrenal insufficiency, and 1 of 15 patients with T1DM developed adrenal insufficiency. The characteristics of the patients who developed multiple endocrine abnormalities are summarized in Supplementary Table S4 (15). There was no consistency in the order or timing of the onset of each endocrine abnormality.

### Association Between the Development of Each Endocrine Abnormality and Survival

**Figure 4.**
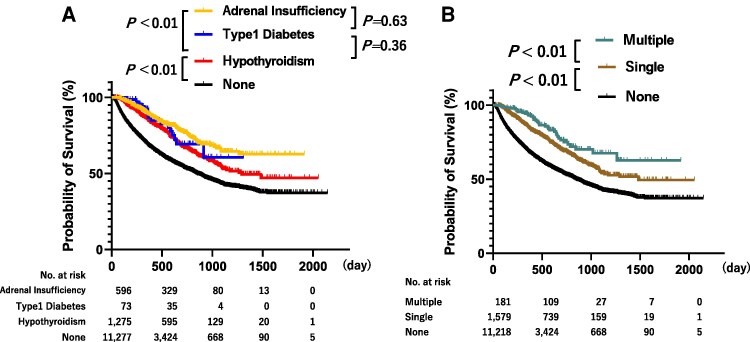
(A) Kaplan–Meier survival curves for patients with each endocrine abnormality. Overall survival was superior in patients who developed adrenal insufficiency, type 1 diabetes, and hypothyroidism compared to patients who did not develop any endocrine abnormalities, (*P* < .01, log-rank test) with 1-year overall survival rates of 89.0%, 86.8%, 85.0%, and 68.6%, respectively (*P* < .01; estimated value). Time represents the number of days elapsed since the first administration. (B) Kaplan–Meier survival curves for patients with single or multiple endocrine abnormalities. The overall survival of the patients who developed multiple endocrine abnormalities was superior to that of the patients who did not develop any, with 1-year overall survival rates of 93.0%, 84.8%, and 68.6%, respectively (*P* < .01, log-rank test) (*P* < .01, estimate). Time represents the number of days since the first administration.

**Figure 5.**
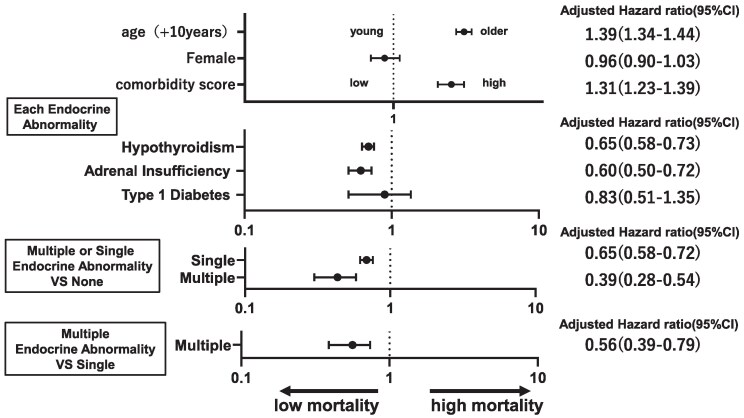
Forest plot of the factors associated with endocrine abnormality and mortality risks determined using Cox regression analysis. Cox regression analysis, adjusted for the number of ICI doses and duration of ICI administration, was performed using age, sex, ICI type, tumor type, prognostic score, and endocrine abnormalities as covariates. ICI-related hypothyroidism and adrenal insufficiency were associated with decreased mortality, and the development of ICI-T1DM was associated with decreased mortality, albeit not significantly. Patients in the single and multiple endocrine abnormality groups exhibited reduced mortality rates compared to those without. Abbreviations: ICI, immune checkpoint inhibitor; irAE, immune-related adverse events; T1DM, type 1 diabetes mellitus.

Among 12 978 patients treated with ICIs, 4738 patients (36.5%) died during the observation period. The median OS of the hypothyroidism and nonhypothyroidism groups was 1291 and 851 days, respectively, whereas patients with adrenal insufficiency and T1DM did not reach the median OS. One-year survival rates of patients with adrenal insufficiency, T1DM, hypothyroidism, and none were 89.0%, 86.8%, 85.0%, and 68.6%, respectively, with better OS in that order. Patients with adrenal insufficiency exhibited better survival than those with hypothyroidism (between hypothyroidism and adrenal insufficiency and between adrenal insufficiency and none: *P* < .01, respectively, log-rank test, Fig. 4A). However, these analyses cannot exclude immortal time bias; therefore, we performed an additional multivariate model adjusted for the number and duration of ICI administrations in additional age, sex, ICI types, and prognostic score. Convincingly, it still showed that the development of hypothyroidism [adjusted hazard ratio (aHR) 0.65; 95% CI, 0.58-0.73, *P* < .01] and adrenal insufficiency was associated with a lower mortality (Fig. 5; aHR 0.60; 95% CI, 0.50-0.72, *P* < .01). A tendency toward lower mortality in T1DM was also observed, although it was not significant (aHR 0.83; 95% CI, 0.51-1.35). After the adjustment, although there was a tendency of decreased mortality in adrenal insufficiency as compared to those with hypothyroidism, there were no significant differences (aHR 0.81; 95% CI, 0.64-1.03, *P* = .09). In addition, we also examined the impact of withdrawal due to causes other than death, and the results were similar (Supplementary Fig. S2) (15).

### Association Between the Development of Multiple Endocrine Abnormalities and Survival

The survival curves of patients with single and multiple endocrine abnormalities are shown in Fig. 4B. The median OS of the single and none groups were 1483 days and 851 days, respectively. One-year OS for the multiple, single, and none groups were 93.0%, 84.8%, and 68.6%, respectively, with better OS in that order (*P* < .01, log-rank test). Furthermore, multivariable analysis with considering the immortal time bias also showed that patients with multiple and single endocrine abnormalities were associated with an improved OS (aHRs 0.39; 95% CI, 0.28-0.54; respectively, both *P* < .01 and aHR 0.65; 95% CI, 0.58-0.72) vs the none group (Fig. 5). In addition, the mortality rate was significantly lower in the multiple group than in the single group (aHR 0.56; 95% CI, 0.39-0.79, *P* < .01). Sensitivity analysis stratified by age groups (≥70 years or not) also demonstrated consistently better survival in patients with multiple and single endocrine abnormalities, in that order, compared with none, and that mortality was significantly lower in the multiple group than in the single group (Supplementary Table S5) (15).

## Discussion

In this study, using a large-scale cohort of >12 000 patients treated with ICI, we demonstrated the precise incidence of multiple endocrine abnormalities associated with ICI. A single endocrine abnormality occurred in 12.0% of the patients and multiple endocrine abnormalities in 1.4%, with the most common combination being hypothyroidism and adrenal insufficiency in 1.3%. We also showed a relatively rare combination of hypothyroidism and T1DM with an incidence of 0.07%; adrenal insufficiency and T1DM with 0.02%; and a triple combination of hypothyroidism, adrenal insufficiency, and T1DM with an incidence of 0.02%. Importantly, we first demonstrated that the development of multiple endocrine abnormalities was associated with better survival in patients treated with ICI after the adjustment for the number and duration of ICI administration.

In this study, patients with multiple endocrine abnormalities demonstrated the best OS in the Kaplan**–**Meier analysis. In addition, the mortality risk with a higher number of endocrine abnormalities, adjusted for the number and duration of ICI administration, was lower (mortality risk compared to the none group: multiple vs single, aHR 0.39 and 0.65, respectively). It is necessary to adjust for immortal time bias because omission of it can lead to inaccurate or misleading conclusions in survival analysis (19). These results indicate that patients with multiple endocrine abnormalities had a better prognosis than those with a single endocrine abnormality. These data are consistent with the direction of ICI-related endocrine abnormalities that do not require pharmacological glucocorticoid treatment (20). ICI therapy can be continued once the patient's general condition stabilizes with replacement therapy. In addition, it is suggested that the development of endocrine abnormalities can be a biomarker of predicting favorable effects of ICI therapy.

Several studies have reported that endocrine abnormalities, such as thyroiditis (21, 22) and hypophysitis (23, 24), are associated with better survival and a preferable response to ICIs in cancer. In contrast, the association between ICI-related T1DM and prognosis has been controversial, with certain reports showing favorable responses and prognosis in a small-scale analysis (25, 26) and another study reporting no association with mortality (27). Moreover, there have been no reports regarding the relative effect on the prognosis of each endocrine irAE and multiple endocrine abnormalities. The low incidence of ICI-related T1DM, especially in multiple endocrine abnormalities, has precluded such an analysis; however, this large-scale cohort study enabled it. We examined data from 1 of the largest cohorts, adjusting for the number and duration of ICI administration, and demonstrated that the mortality rate was lower in those with adrenal insufficiency (aHR 0.60; 95% CI, 0.50-0.72) followed by those with hypothyroidism (aHR 0.65; 95% CI, 0.58-0.73). We previously demonstrated that the development of T1DM was associated with a lower mortality (aHR 0.60; 95% CI, 0.37-0.99) (17). In this study, T1DM was associated with lower mortality (aHR 0.83; 95% CI, 0.51-1.35), but not significantly, probably because of the patient number.

Although the precise mechanisms underlying the association of endocrine irAE with better outcome are still unknown, several mechanisms have been discussed. The strong immune reaction including endocrine irAE may represent bystander effects from activated T-cells, which can lead to a strong antitumor effect (28, 29). One of the other mechanisms is explained by a common antigen between tumor and target tissue, causing a cross-immune reaction (30). Interestingly, it has recently been reported that at least a part of ICI-related hypophysitis can occur as a paraneoplastic syndrome, in which the tumor ectopically expresses pituitary antigens (31, 32).

Since thyroiditis and hypophysitis are often not appropriately diagnosed with the disease names in real-world practice, we defined endocrine abnormalities by using each replacement therapy (levothyroxine, hydrocortisone, and insulin) and the charge for T1DM in ICI-related T1DM. The incidence of endocrine abnormalities except for thyroiditis in this study was comparable with that in a prospective study in Japan, in which the diagnosis was performed by endocrinologists (33), indicating the validity and usefulness of these definitions. Regarding thyroiditis, some cases reveal euthyroid after a thyroiditis phase, indicating that it was underestimated. In terms of adrenal insufficiency, it is difficult to distinguish between primary and secondary adrenal insufficiency; considering previous reports, adrenalitis is far rarer than hypophysitis, and more than 90% of ICI-related adrenal insufficiency is secondarily associated with hypophysitis (34, 35). Therefore, adrenal insufficiency was considered to represent mostly hypophysitis.

In terms of hypothyroidism, most thyroid irAEs present as painless thyroiditis with transient thyrotoxicosis, followed by primary hypothyroidism (21, 36, 37). Over 40% of patients with initial thyrotoxicosis will develop permanent hypothyroidism requiring thyroid hormone replacement therapy (36). Some patients can present with primary hypothyroidism without a diagnosis of the thyrotoxic phase (38). Central hypothyroidism is observed in half of the patients treated with anti-CTLA-4 antibody, although it can be transient (39); therefore, our definition of hypothyroidism may have included such patients. These data indicate that the definition of hypothyroidism requires consideration of the possibilities of heterogeneous endocrine irAEs in this study; however, the information for the necessity of replacement therapy is important, so we believe that these data are clinically relevant.

The incidence of endocrine abnormalities in this study was significantly higher in patients receiving combination therapy of anti-PD-1 and anti-CTLA-4, supporting previous reports (4, 39). Although hypothyroidism was reported to be more common with anti-PD-1 monotherapy (21) and adrenal insufficiency with anti-CTLA-4 monotherapy (4), no such finding was observed in this study, probably because of the definition we used. This may also be because only 0.2% (n = 24) of the patients in this study were treated with anti-CTLA monotherapy, 17.9% (n = 2326) were treated with anti-PD-L1 monotherapy, and 3.0% (n = 394) were treated with sequential therapy of anti-PD-1 and anti-PD-L1 during the observation period. Female sex and combination therapy with anti-PD-1 and anti-CTLA-4 were associated with hypothyroidism, which is consistent with the 2 large previous studies (36, 40). Younger age, male sex, and combination therapy of anti-PD-1 and anti-CTLA-4 were associated with adrenal insufficiency. Although ICI administration is more common in males and 74.6% of patients were male in this study, even after adjusting for sex, male patients were at a high risk for adrenal insufficiency. The development of T1DM was associated with preexisting hypothyroidism, similar to our previous study (17). It has been reported that 17% of patients with ICI-related T1DM have a history of Hashimoto's disease (41), which may be associated with the development of T1DM as an autoimmune predisposition.

Although there have been many case reports of multiple endocrine abnormalities (8-10), the precise incidence of multiple endocrine abnormalities has not been clarified. In this study, we clearly demonstrated that 1 in 7 patients with hypothyroidism will develop adrenal insufficiency, indicating that caution is necessary during follow-up for patients with hypopthyroidism. Furthermore, 1 in 15 patients with T1DM developed adrenal insufficiency, which can be associated with lethal conditions of adrenal crisis (42).

This study had several limitations. The data depended on claims data codes for diseases, drugs, and instructional charges without laboratory values; therefore, a detailed analysis of pathological conditions is difficult. The definitions of thyroiditis are based on the replacement therapy rather than the disease names, indicating that the number of patients with thyroiditis was underestimated because some cases return euthyroid after the thyroiditis; therefore, we used the term endocrine abnormalities rather than ICI-related thyroiditis. In particular, a lack of information on C-peptide and antiglutamic acid decarboxylase antibodies resulted in difficulty in the diagnosis of T1DM; however, we used the prescription of insulin with a specific self-injection fee for T1DM, as this is strictly applied for T1DM. Indeed, it has been shown that the sensitivity was 0.72 and specificity was 1.00 based on this definition for the diagnosis of T1DM in a general population (43). In this respect, patients with mild T1DM without insulin therapy, like latent adult-onset autoimmune diabetes) (44) may be underestimated. In addition, it is difficult to distinguish central and primary insufficiency in the adrenal or thyroid in the definition used in this study; however, we believe that the presence of each replacement therapy is clinically relevant and this problem is a trade-off of patient number and laboratory values in using a large claims database. Furthermore, although the analysis was performed with the adjustment for age, sex, ICI type, number of ICI administrations, duration, cancer type, and comorbidity score, there may still be confounding factors related with death and withdrawal. The use of a sufficient sample size and relatively less biased data allowed us to clarify the precise incidence and association with survival.

In summary, we demonstrated the incidence, risk factors, and association of multiple endocrine abnormalities with OS using a large administrative claims database. The development of multiple endocrine abnormalities was associated with superior survival compared with a single endocrine abnormality in patients treated with ICI.

## Data Availability

The data that support the findings of this study are available from DeSC Healthcare Inc. (Tokyo, Japan); restrictions apply to the availability of these data, which were used under license for the current study and so are not publicly available. However, the data are available from the corresponding author upon reasonable request and with permission from DeSC Healthcare Inc. (Tokyo, Japan).

## Abbreviations

aHR: adjusted hazard ratio
CI: confidence interval
CTLA-4: anti-cytotoxic T-lymphocyte antigen 4
ICI: immune checkpoint inhibitor
irAE: immune-related adverse event
OS: overall survival
OR: odds ratio
PD-1: anti-programmed cell death protein 1
PD-L1: anti-programmed cell death ligand 1
T1DM: type 1 diabetes mellitus
